# Evening Primrose Oil Ameliorates Hyperleptinemia and Reproductive Hormone Disturbances in Obese Female Rats: Impact on Estrus Cyclicity

**DOI:** 10.3389/fendo.2019.00942

**Published:** 2020-01-30

**Authors:** Hebatallah H. Atteia, Sharifa Alzahrani, Nagla A. El-Sherbeeny, Amal M. Youssef, Noha E. Farag, Eman T. Mehanna, Reda Elhawary, Gehan A. Ibrahim, Amr Elmistekawy, Sawsan A. Zaitone

**Affiliations:** ^1^Department of Pharmaceutical Chemistry, Faculty of Pharmacy, University of Tabuk, Tabuk, Saudi Arabia; ^2^Department of Biochemistry, Faculty of Pharmacy, Zagazig University, Zagazig, Egypt; ^3^Pharmacology Department, Faculty of Medicine, University of Tabuk, Tabuk, Saudi Arabia; ^4^Department of Pharmacology, Faculty of Medicine, Suez Canal University, Ismailia, Egypt; ^5^Department of Physiology, Faculty of Medicine, Taibah University, Medina, Saudi Arabia; ^6^Department of Physiology, Faculty of Medicine, Suez Canal University, Ismailia, Egypt; ^7^Department of Biochemistry, Faculty of Pharmacy, Suez Canal University, Ismailia, Egypt; ^8^Department of Pathology, Faculty of Medicine, Al-Azhar University, Cairo, Egypt; ^9^Clinical Pathology Department, Faculty of Medicine, Suez Canal University, Ismailia, Egypt; ^10^Department of Internal Medicine, Gastroenterology Division, Faculty of Medicine, Al-Azhar University, Cairo, Egypt; ^11^Department of Pharmacology and Toxicology, Faculty of Pharmacy, University of Tabuk, Tabuk, Saudi Arabia; ^12^Department of Pharmacology and Toxicology, Faculty of Pharmacy, Suez Canal University, Ismailia, Egypt

**Keywords:** dietary obese female rats, estrus cyclicity, evening primrose oil, hyperleptinemia, reproductive hormone disturbances, insulin resistance

## Abstract

Obesity is a public health burden disturbing all body functions and reproductive hormones. As obesity increases among females, there will be a rising challenge to physicians in care from fertility problems. Evening primrose oil (EPR oil) contains essential fatty acids including omega-6 linoleic acid with strong anti-inflammatory activity. Since EPR oil has utility in alleviating dysmenorrhea, this study aimed to ascertain its modulatory effect on systemic inflammation, reproductive hormones and estrus cycle irregularity in female obese rats. Thirty-two female rats were distributed to 4 groups: (i) normal, (ii) dietary obese-control female rats, and (iii and iv) dietary obese female rats treated with EPR oil (5 or 10 g/kg). Rats were examined for estrus regularity by taking vaginal smears daily during the last 2 weeks of the experiment. Serum level of insulin, leptin, adiponectin, and inflammatory cytokines was measured. In addition, serum lipid profile, and liver enzyme activities were estimated. Adipose tissues were taken for histopathologic examination as well as determination of gene expression for leptin, leptin receptors, adiponectin, and visfatin. Obese rats exhibited significant weight gain (90.69 ± 8.9), irregular prolonged estrus cycles (83.33%), increased serum levels of insulin, leptin, prolactin and testosterone and decreased gonadotropin levels. EPR oil exhibited a curative effect on obesity-related irregularity in estrus cycle and ovarian pathology. The underlying molecular mechanism may be related to reduction of systemic inflammation, alleviating insulin resistance and modulation of adipokine expression. EPR oil may be considered as a promising therapeutic intervention against obesity-related female hormonal disturbances and estrus irregularity.

## Introduction

Clinical observations have indicated a solid association between obesity and infertility in females (1). Obese women suffer from insulin and leptin resistance, inducing hyperandrogenemia, modulation of steroidogenesis and polycystic ovaries syndrome (PCOS) (2–5). Obesity increases systemic inflammation and disturbs the reproductive functions via negative effects on the ovaries (2). The levels of insulin, estrone, luteinizing hormone (LH), and androstenedione are known to increase. These hormonal changes deteriorates hypothalamic-pituitary-gonadal axis leading to different gynecological consequences (6).

Adipose tissue secretes a variety of molecules known as adipokines which regulate many functions. Some of the major adipokines released from adipose tissue are leptin, adiponectin, and visfatin. Adipokines are considered key players in regulating the reproductive function (7).

Leptin is a 16 kD protein consisting of 146 amino acids which is principally secreted by adipose tissue (8) that functions to inhibit feeding (9). Furthermore, it has various effects on the endocrine systems, tissue remodeling, and fertility (10). Leptin influences gonadotropin secretion and ovarian steroidogenesis in human (11) and rat (12). A lack of leptin or leptin receptors has been linked to infertility and delayed puberty development in humans and rodents (13).

On the other hand, adiponectin increases insulin sensitivity and its level declines with obesity and increases with weight loss (14, 15). Adiponectin receptors are widely located in female reproductive tissues, including ovaries, endometrium and placenta (16). It is reported to be beneficial to reproductive function (17). Visfatin is an insulin-mimetic adipokine that promotes the synthesis and storage of fat, regulates glucose and lipid metabolism *in vivo*, Visfatin controls energy metabolism balance and is linked to the reproductive function (18). It was reported to directly correlates with PCOS (19).

Evening primrose oil (EPR oil), derived from *Oenothera biennis* L plant seeds, contains high percentage of essential fatty acids for body health comprising omega-6 linoleic acid (LA) and γ-LA (20). It possesses direct and indirect anti-inflammatory action due to its sterols content (21) or since its γ-LA content acts as a precursor of prostaglandin E1 (22).

In the field of gynecology, many midwives widely suggest EPR oil to accelerate ripening of the cervix in order to reduce the time of labor and the incidence of delayed pregnancies (23). Interestingly, γ-LA has been reported to alleviate premenstrual syndrome and dysmenorrhea (24). Therefore, EPR oil is perhaps a good treatment for these conditions. Previous clinical studies suggested the usefulness of EPR oil in rheumatoid arthritis (25), ulcerative colitis (26) as well as insulin resistance when used in combination with vitamin D (27).

In an attempt to determine the utility of EPR oil in dietary obese female rats, its effect was assessed in terms of controlling systemic insulin resistance and inflammation in addition to modulation of adipokine and gonadotropin levels. The impact of EPR oil on obesity-induced ovarian pathology and estrus cyclicity was also determined. Positive results may suggest EPR oil as a candidate for obesity related PCOS.

## Materials and Methods

### Diet and Treatments

The high fat diet (HFD) is often utilized for induction of obesity in female rats. It was prepared by mixing the standard chow diet (87.7% w/w) with the following fatty components: 10% w/w pork fat, 2% w/w cholesterol and 0.3% w/w bile salts (28, 29). Each gelatin capsule of Primaleve (GlaxoSmithKline) contained 1,000 mg of EPR oil (including 9–10% γ-LA). The oily content of the capsule was withdrawn with a sharp needle of a 3-ml syringe.

### Design of the Experiment

All procedures for animal handling were in accord with the Guideline for the Care and Use of Laboratory Animals published by the US National Institutes of Health (NIH Publication No. 80-23, revised in 1978). All experimental procedures were approved by the Institutional Research Ethics Committee at Faculty of Pharmacy, Suez Canal University [approval number 201605RA2].

**Figure 1:**
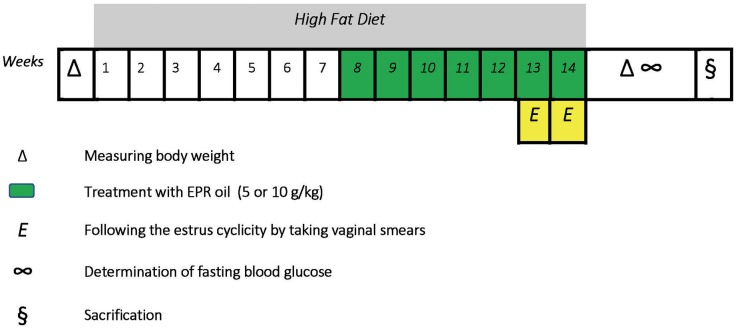
A scheme describes the course of the experiment.

Twenty four female albino Wistar rats were purchased from Moustafa Rashed Company in Saqqarah (Giza, Egypt). The rats aged 8–9 weeks at the beginning of the experiment. Female rats were kept in plastic cages at room temperature (27± 5°C) and normal light-dark phases with unhindered access to food and water. Rats had initial body weights equal 110–142 g. Six rats received standard diet for 14 weeks (WKs), received oral doses of distilled water (10 ml/kg, weeks 8–14) and categorized as a normal group; other rats received HFD for 14 weeks to begin a model of dietary obesity. Rats receiving HFD were divided equally into three groups. The first one received oral doses of distilled water (10 ml/kg, weeks 8–14) and considered the obese control group while the other two groups received oral treatment with EPR oil (5 or 10 g/kg/day, p.o.) (30, 31). Treatment with distilled water or EPR oil started from the beginning of week 8 and continued along with HFD until the end of week 14 (a 7-week therapeutic period). The time-course of the study is shown in Figure 1.

### Detection of Disturbances in Estrus Cyclicity

The percentage of animals with irregular cycles was determined by vaginal smears. The cytology samples were collected daily in the early morning (8:30–9:30 a.m.) for minimum 2 successive cycles (32) by applying a water-moistened cotton bud to be inserted 1 cm intra-vaginally to ensure loading sufficient number of cells on the swab. The removed aqueous fluids were then spread on slides to prepare smears and left at room temperature until completely dried. The prepared smears were immediately immersed in a jar containing 70% alcohol for 1 or 2 min for fixation followed by rapid staining with 0.5% Loba chemie methylene blue solution (Mumbai, India) for 3 min. This was followed by washing with tap water and examined by a light microscope (33, 34). The distinguishing phases of the cycle can be classified in the following as estrus (E), metestrus (M), diestrus (D), and proestrus (P). A typical regular cycle lasts for 4 days. If the of estrus cycle is <4 or >5 days hence, the cycle is marked as not regular (35).

### Blood Glucose Measurement and Collection of Samples

At the end of week 14, body weights of the animals were measured and body weight gain % was calculated relative to the baseline value. After an overnight, fasting blood sugar (FBS) levels were determined using blood samples attained from the tail tip using a blood glucometer. Then, rats were anesthetized and sacrificed by dislocation at the cervical vertebra. Blood samples were taken from the orbital sinus, kept for 20 min at room temperature and centrifuged at 1,600 × g for 8 min. Then, sera were separated and stored at −20°C till the time of analyses. Moreover, the total abdominal white adipose tissues were collected from mesenteric, parametrial, and retroperitoneal tissues and weighed. After that, the adipose tissue index was determined as the percentage of the ratio between adipose tissue weight (g) and whole body weight (g) × 100.

### Determination of Serum Hormone and Cytokine Levels

The insulin level was measured by the SunRed Bio rat insulin ELISA kit (Shanghai, China). Rat estrogen(E) ELISA kit (MBS703614) employs the competitive inhibition enzyme immunoassay technique, a quantitative sandwich rat follicle stimulating hormone (FSH) ELISA kit (MBS017508), a sandwich ELISA kit for rat LH (MBS 2509833), sandwich ELISA for rat prolactin (PRL) (MBS727546) and rat testosterone ELISA kit (MBS262661) were obtained from MyBioSource (San Diego, California, USA). Rat progesterone ELISA kit (CSB-E07282r) employed the competitive enzyme immunoassay technique obtained from CUSABIO (Hubei, China). Serum levels of adiponectin, leptin, interlukin1β (IL1β) and tumor necrosis factor-α (TNF-α) were estimated by CUSABIO rat ELISA kits (8400 Baltimore Avenue, MD, USA) in accordance to the manufacturer's instructions.

### Calculation of the Insulin Resistance Index

For calculation of the homeostasis model assessment of insulin resistance [HOMA-IR] index, the next formula was applied: HOMA-IR index = [FBS mM per L x fasting insulin μU per mL]/22.5 (36).

### Colorimetric Assays for Liver Enzymes and Lipid Profile

Serum level of liver enzyme activities including alanine aminotransferase (ALT) and aspartate aminotransferase (AST) were assayed colorimetrically. Furthermore, serum lipid profile fractions comprising triglycerides (TG), total cholesterol (TC) in addition to low- and high-density lipoprotein–cholesterol (LDL-C, HDL-C) were determined using assay kits from Biodiagnostic (Cairo, Egypt) and an ultraviolet-visible spectrophotometer UNICO 7200 Spectrophotometer (New Jersey, USA).

### Quantitative Real-Time Reverse-Transcription Polymerase Chain Reaction for Adipokines mRNAs in Adipose Tissue

White adipose tissue expression of leptin, leptin receptor, adiponectin, and visfatin was determined by one step quantitative real-time PCR as described previously (37). GAPDH was measured as a housekeeping gene. Table 1 demonstrates the specific primers and annealing temperatures used for each determined gene. Relative quantification of the gene in each sample was estimated using LIVAK method (38).

**Table 1:** Primer sequence for the measured genes.

**Gene**	**Forward primer**	**Reverse primer**	**Annealing temp**
Leptin	5′-GACATTTCACACACGCAGTC−3′	5′-GAGGAGGTCTCGCAGGTT-3′	53°C
Leptin receptor (Ob-Rb)	5′-TGCTCGGAACACTGTTAAT−3′	5′-GAAGAAGAGCAAATATCA-3′	52°C
Adiponectin	5′-AATCCTGCCCAGTCATGAAG-3′	5′- CATCTCCTGGGTCACCCTTA-3′	53°C
Visfatin	5′-CCTTACCTTAGAGTCATTCA−3′	5′-GACATTCTCAATACTCCAC−3′	46°C
GAPDH	5′-ATGACTCTACCCACGGCAAG−3′	5′-GATCTCGCTCCTGGAAGATG-3′	54°C

### Histopathology

Retroperitoneal fats were fixed for 48 h in formalin-alcohol, inserted in paraffin, cut into 3-μm thick specimens and finally hematoxylin and eosin (HE) staining was done. Sections were examined by a CX21 microscope (Olympus, Tokyo, Japan) and images were captured at 20 magnification. Adipocyte space diameters were measured in two different microscopic fields and the average was then calculated.

Furthermore, samples were collected from ovarian tissues and fixation was done in 10% paraformaldehyde for 1 day. After that, sections were dipped in water followed by serial dilutions of alcohol. Sections were then inserted in paraffin wax and were sectioned at 4-μ. The cut tissue specimens were stained by HE or Masson's trichrome and inspected by a light microscope (39) to detect pathological changes. A calibrated digital microscope camera (Tucsen ISH1000, Yuscen Photonics Co. Ltd., China) using Olympus® CX21 microscope, with resolution of 10 megapixels (3,656 × 2,740 pixel for every image). All Masson's trichrome stained sections were captured at original magnification 400x (Objective 40x), UIS optical system (Universal Infinity System, Olympus, Tokyo, Japan).

### Statistics

SPSS 21 software was utilized to explore the obtained results. Quantitative data were represented as means ± SEM. Assessment was completed by one-way ANOVA followed by Bonferroni's test. Chi square test was applied for qualitative data for % of estrus cycle irregularity. All reported data were two-tailed and *P* < 0.05 was set as the level of statistical significance.

## Results

### Dietary Obesity Markers

**Figure 2:**
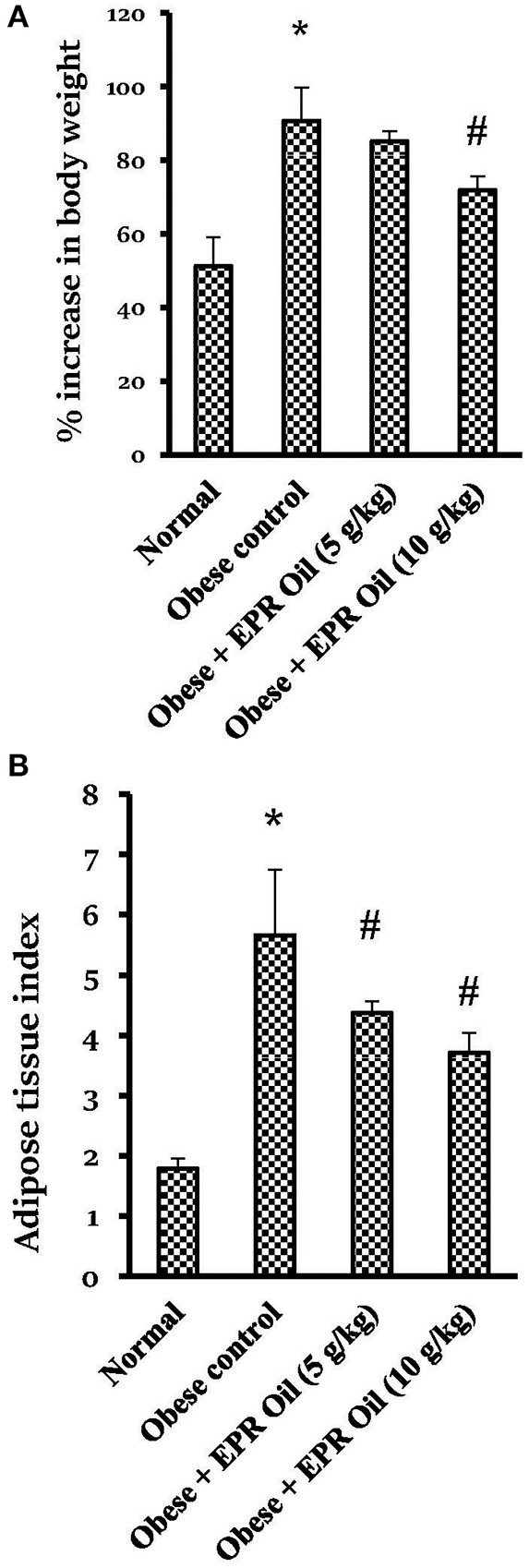
Percent change in body weight and adipose tissue index in the experimental groups. **(A)** % increase in body weight and **(B)** Adipose tissue index. Data are mean ± S.E.M. Bonferroni's test was used to determine differences among individual groups. *Different from normal group, ^#^different from obese control group at *P* < 0.05.

Rats fed with a HFD for 14 weeks showed approximately 91% rise in body weight vs. 51% in the normal group. Treatment with the large dose of EPR oil produced a significant reduction in the body weight gain vs. the obese control group (Figure 2A). Furthermore, there was a 3-fold increase in the calculated adipose tissue index in the obese control female rats vs. the normal females. Therapeutic doses of EPR oil (5 or 10 g/kg) in obese female rats significantly reduced the adipose tissue index vs. the obese female group (Figure 2B).

Serum insulin, FBS and hence HOMA-IR index levels in obese control female rats were greater than the levels reported in normal female rats. EPR oil created dose-dependent improvements in fasting insulin and HOMA-IR index vs. the obese control group (Table 2). Further, the serum levels of the adipokines, leptin, and the inflammatory indicators, TNF-α, and IL1β, were elevated in obese female controls compared to the normal females. Treatment with EPR oil significantly decreased these three markers compared to the obese female group. Conversely, serum adiponectin level was significantly lower in obese rats, with a marked increase upon treatment with EPR oil (10 g/kg) (Table 2).

**Table 2:** Effect of evening primrose oil (5 or 10 g/kg) on serum biochemical parameters in obese female rats.

	**Normal**	**Obese control**	**Obese + EPR oil** **(5 g/kg)**	**Obese + EPR oil** **(10 g/kg)**
FBS (mg/dL)	74.11 ± 1.32	99.33 ± 1.12^*^	87.01 ± 0.90^*^^#^	80.21 ± 0.89^#^
Insulin (μIU/L)	9.14 ± 1.13	42.22 ± 1.23^*^	34.02 ± 0.78^*^^#^	23.14 ± 1.12^*^^#^^$^
HOMA-IR index	1.69 ± 0.34	10.33 ± 1.12^*^	7.14 ± 1.04^*^^#^	4.55 ± 1.30^*^^#^^$^
TG (mg/dL)	144.12 ± 2.67	224.14 ± 3.34^*^	179.12 ± 2.12^*^^#^	165.22 ± 2.00^*^^#^
TC (mg/dL)	87.21 ± 2.10	130.23 ± 2.99^*^	122.23 ± 2.21^*^	125.12 ± 2.33^*^
LDL-C (mg/dL)	57.22 ± 1.12	71.25 ± 1.23^*^	66.13 ± 1.87^*^	68.12 ± 1.08^*^
HDL-C (mg/dL)	42.12 ± 1.55	29.34 ± 1.67^*^	32.12 ± 1.21^*^	32.01 ± 1.11^*^
ALT (IU/L)	48.22 ± 2.12	80.12 ± 2.33^*^	72.34 ± 3.12^*^	73.12 ± 3.04^*^
AST (IU/L)	68.21 ± 4.12	88.23 ± 5.12^*^	84.45 ± 4.22^*^	85.12 ± 3.98^*^
Leptin(ng/mL)	2.00 ± 0.30	5.50 ± 0.70^*^	3.80 ± 0.40	2.70 ± 0.30^#^
Adiponectin (ng/mL)	3.20 ± 0.80	1.30 ± 0.20^*^	1.90 ± 0.35	2.50 ± 0.50^#^
TNF-α (pg/mL)	24.67 ± 3.50	50.50 ± 5.77^*^	38.50 ± 3.89	32.30 ± 3.33^#^
IL1β (pg/mL)	20.17 ± 3.33	55.17 ± 4.47^*^	42.60 ± 4.20^*^	30.20 ± 3.90^#^

*EPR oil, evening primrose oil; FBS, fasting blood sugar; HOMA-IR, homeostasis model assessment of insulin resistance; TG, triglycerides; TC, total cholesterol; LDL-C, low-density lipoprotein–cholesterol; HDL-C, high-density lipoprotein–cholesterol; ALT, alanine aminotransferase; AST, aspartate aminotransferase. Data are represented as mean ± SEM. Comparisons were performed by one-way ANOVA followed by Bonferroni's test for multiple comparisons*.

* *Significantly different from Normal group*,

# *significantly different from Obese control group*,

$ *significantly different from obese + EPR oil (5 g/kg) group at p < 0.05*.

Table 2 demonstrates that obese control group showed greater serum TG, TC, LDL-C, and lower HDL-C vs. the normal group. EPR oil (5 or 10 g/kg) equally and significantly lessened the high serum TG level without effect on the other parameters. Serum ALT and AST increased in obese female group vs. the normal female group. EPR oil did not produce decreases in serum levels of these enzymes (Table 2).

**Figure 3:**
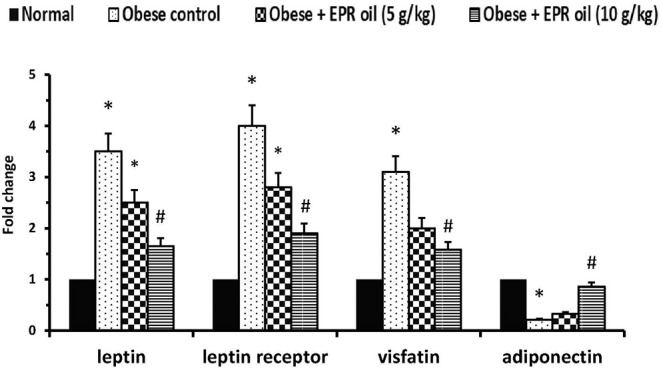
Polymerase chain reaction for adipokines in the experimental groups. Data are mean ± S.E.M. Bonferroni's test was used to determine differences among individual groups. *Different from normal group, ^#^different from obese control group at *P* < 0.05.

Adipose tissue expression of leptin and leptin receptors was increased in obese control rats (≈3.5- and 4-fold increases, respectively) vs. the expression levels in the normal group. A similar increase (≈3.1-fold) was detected in mRNA expression of visfatin; however, a decrease was detected in the expression of adiponectin. Treatment with the large dose of EPR oil ameliorated the aforementioned changes vs. the obese control group. The low dose of EPR oil failed to produce a similar effect (Figure 3).

**Figure 4:**
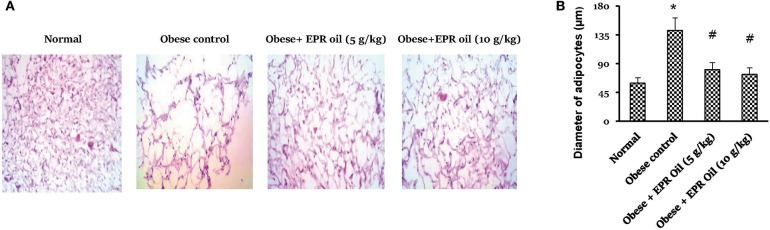
Diameter of adipocyte spaces in white adipose tissue. **(A)** Photographs for adipose tissue specimens stained with hematoxylin and eosin showing different adipocyte spaces in the study group. **(B)** Column chart showing mean value for adipocyte space diameters. Data are mean ± S.E.M. Bonferroni's test was used to determine differences among individual groups. *Different from normal group, ^#^different from obese control group at *P* < 0.05.

Figure 4 shows the adipocyte space diameters in retroperitoneal adipose tissue section which was significantly larger in obese female rats vs. the measured diameters in normal rats. EPR oil (5 or 10 g/kg) equally and significantly reduced the adipocyte diameters.

### Estrus Cycles and Sex Hormone Levels

**Figure 5:**
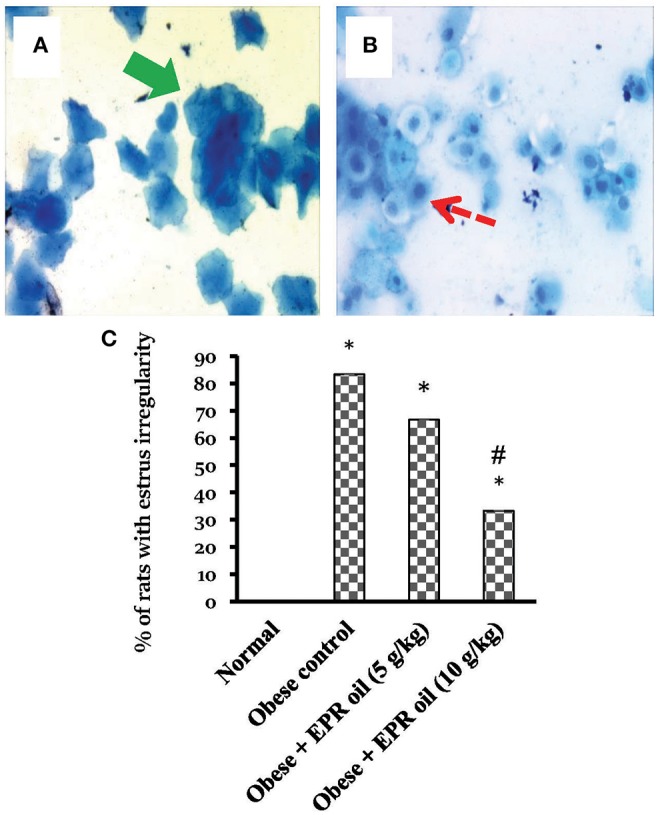
Cell types in vaginal smears and percentages of rats with estrus cycle irregularities. Photos represent the squamous cornified epithelial cells (Thick green arrow) **(A)**, the nucleated epithelial cells (dashed red arrow) **(B)**. The presence or absence of specific cell types indicates the estrus stage estrus, metestrus, diestrus, and proestrus cycle. Methylene blue ×40. **(C)** Percentage of rats with irregular cycles in the experimental groups. Irregularity of estrus cycle in female rats was tested using vaginal smears for a period of 2 weeks. Data are presented as % of irregular cycles (out of 6 female rats). Chi square test was applied at *P* < 0.05. EPR oil: evening primrose oil. *Different from normal rats, ^#^different from obese control rats.

In the vaginal smear, each phase can be primarily identified according to the percentage of types of 3 primary cells present. These types include nucleated epithelial cells, cornified squamous epithelial cells, and leukocytes (Figures 5A,B). Numerous cornified cells were generally observed as clumps and sheets in estrus stage. On the other hand, metestrus stage consists completely of great number of leukocytes and few big non-nucleated epithelial cells. Diestrus stage contains chiefly leukocytes along with large numbers of epithelial cells. Furthermore, proestrus stage is characterized by nucleated epithelial cells. Importantly, obese female rats showed high percentage of estrus irregularity (Figure 5C). A considerable fraction of female rats have 6–7 days cycles. The prolonged cycles were detected in form of existence of a 2nd or 3rd day of a couple of the 4 stages. The commonly prolonged stages were the diestrus and estrus.

**Figure 6:**
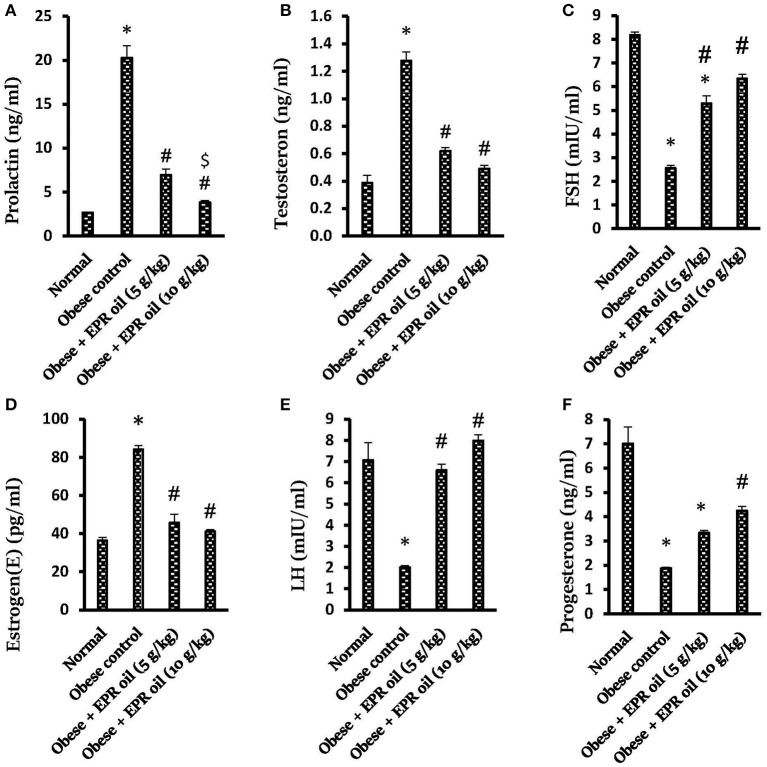
Effect of evening primrose oil (5 or 10 g/kg) on serum hormone levels in obese female rats. **(A)** Prolactin, **(B)** Testosterone, **(C)** FSH, **(D)** Estrogen(E), **(E)** LH and **(F)** Progesterone levels. Data are mean ± S.E.M. Bonferroni's test was used to determine differences among individual groups. *Different from normal group, ^#^significantly different from obese control group, ^$^different from obese + EPR oil (5 g/kg) group at *P* < 0.05. EPR oil, evening primrose oil; LH, luteinizing hormone; FSH, follicle stimulating hormone; PRL, prolactin.

Moreover, obese control female rats showed hyperprolactinemia and greater serum testosterone and estrogen levels compared to normal female rats. In contrast, serum progesterone, FSH and LH levels were lower compared to the normal female rats. Treatment with both doses of EPR oil (5 or 10 g/kg) significantly ameliorated the serum levels of these hormones vs. the obese control females (Figure 6).

**Figure 7:**
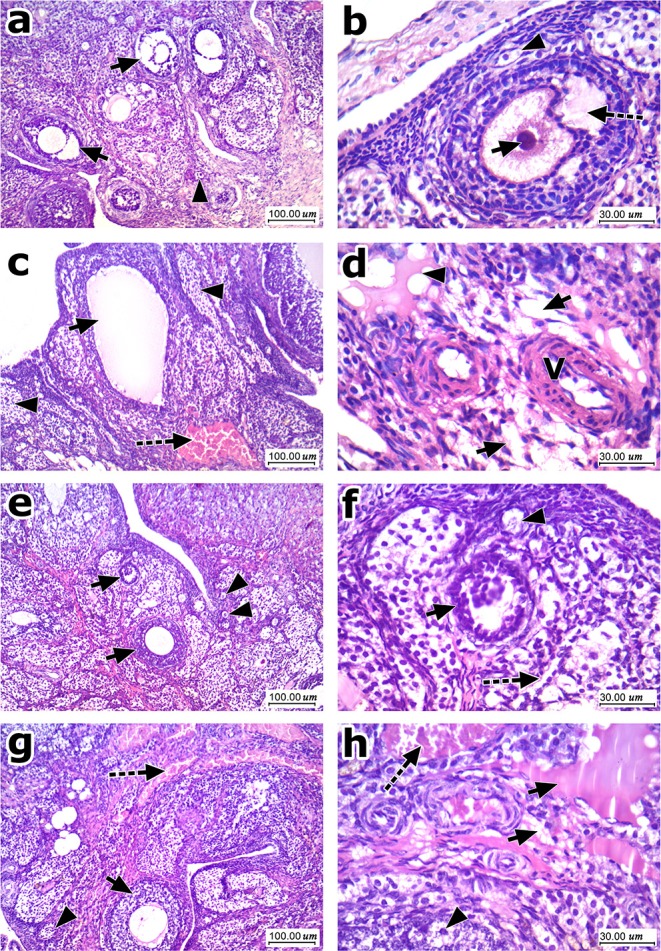
Histopathologic features of ovarian sections stained with hematoxylin and eosin. **(a)** Low magnification of ovarian cortex of *normal* group showed many mature Graafian follicles (arrow) with many primordial follicles (arrowhead), stroma in-between formed of spindle cells and vessels. **(b)** High magnification showed maturing follicle (Antral) with ovum (arrow) in the center surrounded by granulosa cells with antral space (dashed arrow), and a nearby primordial follicle (arrowhead). **(c)** Low magnification of ovarian cortex of control obese group showed one of the cysts (arrow), atretic follicles (arrowhead), and marked congestion (dashed arrow). **(d)** High magnification showed marked stromal edema (arrow) with cystification (arrowhead) and scattered vessels showing slight thickening of their walls (V). **(e)** Low magnification of ovarian cortex of EPR oil (5 g/kg) treated group showed marked improvement, no cysts presented, multiple numerous primordial follicles (arrowhead) detected, and secondary follicles (arrow). **(f)** High magnification showed secondary follicles (arrow), primordial follicles (arrowhead) and residual mild stroma edema (dashed arrow). **(g)** Low magnification of ovarian cortex of EPR oil (10 g/kg) treated group showed moderate improvement, residual congested vessels (dashed arrow) secondary follicles (arrow), and scattered atretic follicles (arrowhead). **(h)** High magnification showed primordial follicles (arrowhead) with residual stromal edema (arrow) and mild congestion (dashed arrow), H&E, 100x, and 400x.

Figure 7 shows histopathologic features of HE stained ovarian sections. Ovarian cortices from the normal group contained many mature Graafian follicles with many primordial follicles, stroma in-between formed of spindle cells and vessels. Mature Graafian follicles contained ova in the center surrounded by granulosa cells, antral space, and a nearby primordial follicle (Figures 7a,b). Ovarian cortices from the obese control group showed cysts, atretic follicles and congested dilated vessels. Marked stroma edema with cystification was prominent at high power fields (Figures 7c,d). Ovarian cortices from all obese rats treated with EPR oil (5 g/kg) showed marked improvement, no cysts, multiple numerous primordial follicles detected and secondary follicles. Secondary follicles, primordial follicles and residual edema were shown (Figures 7e,f). Ovarian cortices from obese rats treated with EPR oil (10 g/kg) showed mild to moderate improvement, residual congested vessels, secondary follicles and scattered atretic follicles. Some primordial follicles were shown with residual minimal edema and mild congestion (Figures 7g,h).

**Figure 8:**
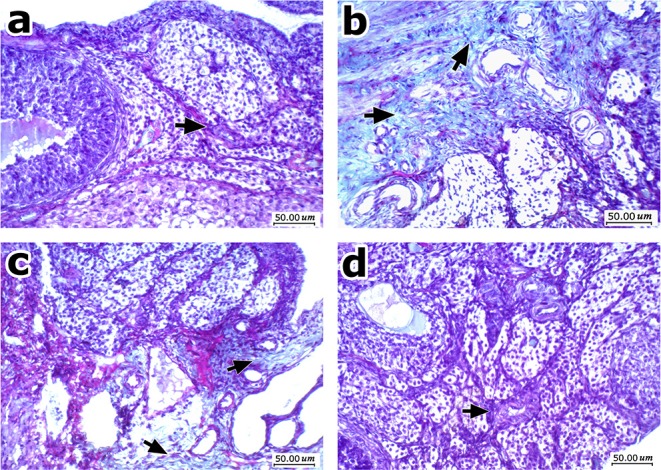
Histopathologic features of ovarian sections stained with Masson's trichrome stain. **(a)** Normal ovary shows ovarian cellular stroma with scattered small thin vessels (arrow) and no fibrosis. **(b)** An image from *obese* control group showing moderate fibrosis and deposition of thick collagen fibers stained green, with more evident perivascular arrangement. **(c)** An image from obese rats treated with EPR oil (5 g/kg) showing improvement with mild residual fibrosis mainly perivascular. **(d)** An image from obese rats treated with EPR oil (10 g/kg) showing improvement with cellular ovarian stroma without perivascular fibrosis, with scattered small vessels (arrow), Masson's trichrome stain, 200x.

Figure 8 shows Masson's trichrome stained ovarian sections. High degree of fibrosis was shown in ovaries from obese control rats (Figures 8a,b). Treatment with EPR oil (5 or 10 g/kg) decreased the amount of fibrotic tissues shown in ovaries (Figures 8c,d).

## Discussion

This study explored -for the first time- the effect of EPR oil on obesity related estrus irregularity, hormonal disturbances, and pathologic changes affecting the genital system. Since EPR oil is already invited to be used in the field of gynecology, it would be acceptable to suggest such remedy for future investigations on obese females with fertility problems.

In the current study, feeding female rats with a HFD led to significant weight gain, adiposity, and increased adipocyte diameters. Serologic investigations indicated hyperglycemia, hyperinsulinemia, insulin resistance in addition to increased lipids and inflammatory cytokines. Comparable results were found previously in rats fed the same diet for 14–16 weeks (31, 40). One human study conveyed a relationship between weight increases and resistance of tissue to insulin (41).

It is well-documented that excess adipose tissue leads to reproductive dysfunction. In females, obesity is linked to menstrual disorder, infertility, and obstetric compilations (17) as well as increased adipokine secretion and modulation of reproduction (42). One of the most common obesity-associated female reproductive disorders is PCOS which is characterized by overweight, amenorrhea and anovulation (43).

In the present study, female rats fed with a HFD showed hyperleptinemia and upregulation of the adipose tissue expression of leptin and the long isoform of leptin receptors. Similar results were obtained earlier by many research groups (28, 44). Leptin is in a direct relation with obesity and insulin resistance while adiponectin level is in an inverse correlation (45). High caloric intakes increase leptin which by turn suppresses appetite and improves thermogenesis in a central mechanism (46).

In relation to fertility, leptin was reported to enhance gonadotropin releasing hormone (GnRH) production via kisspeptin neurons located in arcuate nucleus of hypothalamus (47), thus plays an important role in initiation of puberty (48). Mice deficient in leptin or leptin receptors display low LH level and partially developed reproductive organs. In agreement with the previous findings, exogenous administration of leptin to ob/ob mice stimulates pubertal development and reproductive organ maturation, increases secretion of LH and restores fertility. Consequently, this explains the role of leptin signaling in female reproductive function (13). Moreover, mice and humans missing leptin receptor were reported to acquire hypothalamic hypogonadism, which delays puberty and results in infertility. In addition to the female circumstance, testicular atrophy and compromised spermatogenesis were found in male mice deficient in leptin signaling (49). However, in most dietary obesity rodent models, hyperleptinemia was documented and explained by occurrence of systemic leptin resistance and reduced physiologic action of leptin (50–52); this may help to describe the affected LH and gonadotropin level in our rat model.

The relationship between hyperleptinemia and female infertility was highlighted in some studies. One study documented that leptin-transgenic mice with 10-fold higher peripheral concentrations of leptin than controls begin to gain excessive weight and develop hypothalamic hypogonadism when their age reaches 20 week (53). Another study on HFD female DBA/2J mice proposed that obesity-linked hyperleptinemia, slowly prompted central leptin resistance, amplified hypothalamic neuropeptide Y transmission and eventually led to hypothalamic hypogonadism (1). Our study demonstrated that treatment with EPR oil mitigated hyperleptinemia and corrected the serum LH and gonadotropin levels.

In the present experiment, female rats fed with a HFD showed lower serum adiponectin level and downregulation of the expression of adiponectin gene in adipose tissues. Adiponectin has been shown to suppress LH and GnRH release (54, 55), representing its putative action in the regulation of hypothalamo-pituitary-gonadal axis (56). Increments in adiponectin serum level were observed in females treated with human chorionic gonadotropin during an *in vitro* fertilization (57); this highlights the action of adiponectin in regulation of the central reproductive endocrine axis (56). Consistently, women with PCOS show decreased high molecular weight adiponectin independent of their body mass index (58). These previous data agree with our findings as EPR oil enhanced serum adiponectin level and boosted serum gonadotropin level.

Additionally, female obese rats in the current study showed high circulating testosterone, estrogen, and PRL levels, with significant decrease of progesterone, FSH and LH levels. Clinical data showed that obesity was considered as a major cause of estrogen/progesterone imbalance (59) and low pituitary production of LH and FSH (60). A smart *in vitro* study in granulosa cells from an ovum from a polycystic ovary highlighted that insulin encourages formation of estradiol in cultured human granulosa cells (61).

In support to the aforementioned evidences, obese women usually suffer from hyperleptinemia, hyperandrogenemia, amplified the aromatization of androgens to estrogens in peripheral tissues, changed secretion of gonadotrophin, declined sex hormone binding globulin, and changed neuroregulation of the hypothalamic-pituitary-gonadal axis.

Moreover, high levels of circulating androgen are mutual characters in obese women, if amenorrhea is present (5, 62). Androgens were thought to produce arrest of follicular maturation.

Regarding PRL, it was found high in women suffering from obesity (63) and high serum PRL levels were linked to insulin resistance and increased HOMA-IR index in both men and women (64, 65). PRL by turn looks to impact ovarian function and long-term hyperprolactinemia is known to hinder the hypothalamus-pituitary axis and endorse anovulatory cycles (26). Collectively, these have been assumed to contribute to the subsequent events leading to discrepancies in the ovulatory process (66).

Indeed, clinical data highlighted a direct relation between circulating visfatin level and visceral adiposity (67). A different research indicated that visfatin is in a direct correlation with inflammation (68). Our findings agree with the aforementioned examples from the literature; obese rats showed upregulated expression of visfatin in adipose tissues and greater serum inflammatory cytokines. Rats treated with EPR oil showed less visfatin expression and low serum level of the inflammatory cytokines.

In the current experiment, obesity was associated with ovarian histopathologic changes like marked stromal edema, cystification, congestion, and thickening of blood vessels. Follicle formation is a highly coordinated process that is influenced by many factors. Studies highlighted many hormones, growth factor and cytokines in the ovary playing crucial roles in the follicular development. The follicles grow and develop orderly under the control of endocrine hormone (69, 70), growth factor precisely produced at different time points (71, 72) and the cytokines associated with the development of follicles include TNF-α, IL1β, and interferon-γ (73–75). The impact of adiposity has been involved in troubles of female reproduction (1). Histological assessment of ovaries from female rats nourished with a cafeteria diet revealed low amount of preantral follicles compared to lean females (76). Moreover, obesity and insulin resistance were linked to ovarian dysfunction in female experimental animals (77). Studies indicate that ovarian high leptin levels may impede the progress of dominant follicles and oocyte maturation (78) and leptin appears to antagonize the ovarian cell stimulation (11). Hence, hyperleptinemia in obese female rats was documented to play a crucial effect in regulating fertility (79).

Beside the ovarian changes, our obese female rats showed disturbances in estrus cyclicity in form of prolongation of cycle days. In agreement with the current results, experimental studies indicated that gestational, and/or post-natal feeding of adult female rodents fed on HFD were reported to enhance pubertal maturity onset and induce estrus cycle irregularity (80, 81). Studies indicated that obesity also reduce implantation (82) and pregnancy rates, induce irregular menses and increase complications with PCOS (83). Interestingly, data from clinical studies provided similar conclusions. For instance, overweight women exhibited greater prevalence of anovulation and menstrual dysfunction (84) and increased risk for poor reproductive function, infertility, conception and pregnancy complications (85). Conversely, weight loss improved reproductive outcomes in these women (86).

EPR oil reduced weight gain and adiposity and favorably improved the elevated insulin resistance seen in female rats fed with HFD. This was accompanied with favorable effects on serum leptin, adiponectin and TG in addition to lessening of serum inflammatory markers, TNF-α, and IL1β. Additionally, EPR oil regulated the estrus cyclicity and improved the histopathologic features seen in the ovaries.

Overall, EPR oil has a strong record of anti-inflammatory activity that suggested its use in many inflammatory conditions including dysmenorrhea. Montserrat-de la Paz et al. (87) considered EPR oil as a source of a new nutraceutical value since it is sometimes used for treating diabetic neuropathy, arthritic and rheumatic conditions and premenstrual/menopausal syndrome (88). The same authors published another article and concluded that long-chain fatty alcohols from EPR oil prevent inflammation in macrophages (87). EPR oil was also suggested to abrogate the prothrombotic adverse effects of celecoxib (31, 89).

The favorable effects of EPR oil are assumed to be linked to the contented γ-LA, since ω-6 fatty acids are used in the 1-series anti-inflammatory eicosanoids synthesis and employ a suppressor effect on leukotriene synthesis (90). The lipid profile from EPR oil showed 7% oleic, 74% LA, and 9% γ-LA and its beneficial properties are related to the direct effect of its content of essential fatty acids (91). Supplementation with EPR oil increases plasma level of γ-LA and its metabolite dihomo-γ-LA. Detailed mechanism is summarized in that this compound is oxidized by 5-lipoxygenase to 15-hydroxyeicosatrienoic acid (15-HETrE) or under the influence of cyclooxygenase, dihomo-γ-LA is converted to series 1 prostaglandins; these molecules possess anti-inflammatory characters. Furthermore, 15-HETrE is able to block arachidonic acid conversion to leukotriene A4 by an inhibitory action on 5-lipoxygenase (92). Overall, since the structure of EPR oil is well-known and all contented acids are known for their anti-inflammatory properties and no previous documentation for any possible anorexic effect was highlighted before. Remarkably, the low dose of EPR oil did not produce any significant effect on body weight but improved insulin resistance and reduced IL1β level beside amelioration of serum PRL, testosterone and gonadotropins. Therefore, we suppose the current action of EPR oil in alleviating obesity related complications is dependent on its anti-inflammatory properties rather than any anticipated effect on food intake.

Additionally, EPR oil was considered to reduce the severity of cyclical mastalgia, a state that is caused mainly by hormonal imbalance in premenopausal women (93). Oral administration of EPR oil was also reported to have an effect against menopausal hot flashes (94). In addition, one study demonstrated that dietary-EPR oil modifies the nociceptive reaction and symptoms accompanying intermittent cold stress and reduces inflammation (88). In animals fed hyperlipeidimic diet, dietary supplementation with *O. biennis* was mentioned to improve the endothelial antithrombotic ability, decrease sub-endothelial thrombogenicity and reduce vascular wall lesions (95). *O. biennis* was found to be effective in the treatment of inflammatory bowel disease (96).

## Conclusions

In the current study, EPR oil enhanced most of the metabolic and endocrine impact of feeding with HFD in female rats. The most genuine effect was the improving of estrus cyclicity and eliminating the changes in ovarian pathology. Importantly, EPR oil should be tested in other models of PCOS to ensure a direct improving effect on ovarian structures and to ensure data obtained in the currents study are not simply due to improving insulin resistance. Future studies are warranted to test the protective effect of EPR oil on other models of chemically induced PCOS to ensure whether the protective effect is based on anti-inflammatory properties and to firmly exclude any anticipated anorexic effect in the current study.

## Data Availability Statement

All datasets generated for this study are included in the article/supplementary material.

## Ethics Statement

The animal study was reviewed and approved by Research ethics committee at Faculty of Pharmacy, Suez Canal University, Ismailia, Egypt.

## Author Contributions

HA, SA, NE-S, NF, AY, and SZ: conceived and designed research. HA, SZ, and EM: conducted animal experiments and analyzed data. EM and GI: performed blood tests. EM and HA: PCR analysis and biochemical assays. RE: histopathological examination. All authors: contributed to reagents or analytical tools, wrote, read, and approved the manuscript.

### Conflict of Interest

The authors declare that the research was conducted in the absence of any commercial or financial relationships that could be construed as a potential conflict of interest.

## Abbreviations

ALT: alanine aminotransferase
AST: aspartate aminotransferase
ELISA: enzyme-linked immunosorbent assay
EPR oil: evening-primrose oil
FSH: follicle stimulating hormone
HDL: high-density lipoprotein–cholesterol
HFD: high fat diet
IL1β: interlukin1β
MOMA-IR index: the homeostasis model assessment of insulin resistance index
LA: linoleic acid
γ-LA: γ-Linoleic acid
LDL-C: low-density lipoprotein–cholesterol
LH: luteinizing hormone
PCOS: polycystic ovaries syndrome
PRL: prolactin
WK: week
TG: triglycerides
TC: total cholesterol
TNF-α: tumor necrosis factor-α.
